# Case control study on risk factors associated with esophageal cancer in Yemen

**DOI:** 10.1186/1471-2458-12-S2-A11

**Published:** 2012-11-27

**Authors:** Al-abed Ali Ahmed Al-abed, Azmi Mohd Tamil, Sami Abdo Radman Al-Dubai

**Affiliations:** 1 Community Health Department, Universiti Kebangsaan Malaysia Medical Centre, Jalan Yaacob Latiff, 56000 Kuala Lumpur, Malaysia; 2 Department of Community Medicine, International Medical School, Management and Science University, Off Persiaran Olahraga, 40100 Shah Alam, Malaysia

## Background

Esophageal cancer is the seventh most common malignancy worldwide and the fifth in the developing countries. Its incidence is rising more rapidly than any other cancer. The risk of developing esophageal cancer between countries may be influenced by diversity of cultures and customs. One of the unique customs in Yemen is chewing khat, therefore the aim of this study was to determine the risk factors associated with esophageal cancer in Yemen, including chewing khat.

## Materials and methods

A hospital-based, unmatched case-control study was conducted in Aljomhory Hospital-Sana’a, Yemen. Forty nine cases of esophageal cancer from Oncology centre and 49 controls from medical ward were included in this study. All participants were interviewed by using a validated questionnaire. Convenient sampling was used to select participants during the period from December 2009 until March 2009.

## Results

There were more elderly patients (> 50 years old) among the cases (75.5%) compared to the controls (26.5%). Chewing khat (OR=7.2, 95% CI 2.43-21.17, P<0.0001) and duration of chewing khat (Z= 6.18, P < 0.001) showed significant association with esophageal cancer. Similar findings was found for smoking and smoking duration (OR= 2.56, 95% CI 1.12-5.86, P= 0.024) and (Z= 6.18, P= < 0.001) respectively. Other factors associated with esophageal cancer were residing in the rural area (OR= 9.16, 95% CI 3.59-23.41, P <0.001), age of more than 50 years old (OR= 8.54, 95% CI 3.44-21.19, P < 0.001) and low education level (OR= 13.7, 95% CI 2.8-67.3), P< 0.001). In multiple logistic analysis, esophageal cancer was associated with advancing age, residing in the rural area, longer duration of chewing khat and longer duration of smoking (p<0.05).

## Conclusions

Advancing age, residing in the rural area, longer duration of chewing khat and smoking in years were associated with higher risk of developing esophageal cancer. Chewing khat for long duration was the most important predictor for esophageal cancer in this study.

